# Support, Patience, and Expectancy: Therapeutic Options Alongside Intensified Treatments for Depression?

**DOI:** 10.1177/070674371405900701

**Published:** 2014-07

**Authors:** Scott B Patten

**Affiliations:** 1 Editor-in-Chief Designate, *The Canadian Journal of Psychiatry*, Ottawa, Ontario; Professor, Departments of Community Health Sciences and Psychiatry, University of Calgary, Calgary, Alberta; Member, Hotchkiss Brain Institute, University of Calgary, Calgary, Alberta.

**Keywords:** depression, depressive disorder, antidepressants, treatment-resistant depression

In addition to a scoping review by Dr Rachelle Ashcroft and colleagues^1^ describing incentives and disincentives for depression treatment and a sex-specific analysis of depressive symptoms in members of the Canadian Armed Forces by Dr Jitender Sareen and colleagues (see Erickson et al^2^), this issue of *The Canadian Journal of Psychiatry* includes 2 Original Research papers on treatment-resistant depression (TRD).^3^,^4^ For the first of these, Ms Sakina Rizvi and colleagues^3^ collected data in primary care settings in Canada. An estimated 21.7% of depressed patients in primary care practices were found to have TRD. The authors point out that their estimate is slightly lower than one arising from the Sequenced Treatment Alternatives to Relieve Depression (commonly referred to as the STAR*D) trial. However, both estimates are very high when compared with community populations. In epidemiologic studies, many new-onset episodes are brief, lasting only a few weeks, even though they are often untreated. The TRD episodes described by Ms Rizvi and colleagues had a mean duration of 36 months (12 months in non-TRD episodes), and they report that more than one-half of these patients were taking multiple psychotropics.

Epidemiologically, this is not surprising. Roughly speaking, prevalence is equal to the product of incidence (the rate of new onset) and duration. Therefore, long-standing episodes will accumulate in the population and can be expected to comprise a much larger proportion of prevalent than incident cases. Patients with long-standing episodes will tend to concentrate in clinical settings because they are more likely to seek treatment and more likely to be engaged in those services for longer periods of time.

As pointed out by Ms Rizvi and colleagues,^3^ guidelines generally call for stepwise implementation of increasingly intensive treatment when more basic approaches appear to fail (addition of psychotherapy is also an option, albeit one that is not evaluated in their study). When an episode is treatment-resistant, the strategy of systematically changing or intensifying treatment seems logical. However, for truly recalcitrant episodes, this approach will become unproductive, leading only to a greater burden of adverse medication effects. Indeed, Ms Rizvi and colleagues describe a considerable burden of such adverse effects in patients with TRD. Conversely, systematic pursuit of new medication trials, and augmentation or combination treatments, may lead to earlier remission. Ms Rizvi and colleagues’ data argue that such steps are being taken more slowly in primary care than guidelines would recommend. Unfortunately, there is no certain way to anticipate resistance or recalcitrance to treatment. Much trial and error is the inevitable result.

Dr Gordon Parker, Dr Rebecca Graham, and Ms Elizabeth Sheppard^4^ explore a possibility that might have helped to resolve this conundrum. Perhaps TRD in nonmelancholic patients is not so much a resistance to pharmacologic treatment (thereby calling for medication changes or more intensive pharmacotherapy) but an example of pursuing the wrong paradigm of treatment in the first place. They posit that nonmelancholic patients with TRD may benefit preferentially from psychotherapy. In this situation, apparent resistance to treatment may really mean that the treatment was a poor match for the patients’ needs. Unfortunately, this hypothesis is not supported by their data. In a comparison of patients with nonmelancholic TRD receiving psychotherapy to those declining participation in such treatment, no significant differences in outcome were observed. As these authors point out, their study does not provide the final word on this question. Their study was not randomized and the sample size was small.

However, it is interesting to note that Dr Parker and colleagues’ results are not entirely negative. The lack of difference in outcome was not because neither group improved. Conversely, both groups improved substantially. Be it the (sometimes) healing powers of time, the resilience of those affected, the episodic nature of depression, or the occurrence of statistical regression to the mean, these patients did improve. In addition to the choice of progressively more intensive pharmacologic treatment or altering the approach entirely, these results serve as a reminder that support, patience, and expectancy are options that are also on the table.
